# Digital tools for assessing bipolar disorder: A scoping review of the current landscape

**DOI:** 10.1016/j.nsa.2026.107004

**Published:** 2026-04-21

**Authors:** Samantha Jacobson, Hannah Carling, Lisa Sarraf, Ethan Draper, Sarah Jacobson, Mélodie St-James, Chris Misiasz, Sianna Williamson, Elisabeth Thibaudeau, Geneviève Sauvé, Katie M. Lavigne, Delphine Raucher-Chéné

**Affiliations:** a Douglas Research Centre, Montreal, QC, Canada; b Faculty of Medicine and Health Sciences, Université de Sherbrooke, Sherbrooke, QC, Canada; c School and Clinical Child Psychology, University of Toronto, Toronto, ON, Canada; d Department of Psychology, Carleton University, Ottawa, ON, Canada; e Department of Bioengineering, Imperial College London, London, UK; f Department of Psychology, McGill University, Montreal, QC, Canada; g Department of Neuroscience, McGill University, Montreal, QC, Canada; h Department of Kinesiology and Physical Education, McGill University, Montreal, QC, Canada; i School of Psychology, Laval University, Quebec, Canada; j Department of Education and Pedagogy, Université du Québec à Montréal, Quebec, Canada; k Department of Psychiatry, McGill University, Montreal, QC, Canada

**Keywords:** Bipolar disorder, Digital monitoring, Wearable sensors, Smartphone apps, Remote assessment

## Abstract

This scoping review synthesizes current evidence on the use of digital tools to assess bipolar disorder across clinical, behavioral, physiological, and psychosocial domains. An electronic search of Ovid, CINAHL, ClinicalTrials.gov, and OpenGrey, completed in March 2026, yielded 374 articles for full-text evaluation. Of which 214 studies met the inclusion criteria and were included in the final review. Most studies were conducted in the Global North, particularly the United States, Denmark, the United Kingdom, and Germany. Participant samples varied widely, although cohorts were typically comprised of young adults and predominantly female, with implications for the contexts in which digital markers have been evaluated across illness stages and demographic groups, and for the extent to which these findings can be generalized. Across studies, mood, sleep, and activity were the most frequently assessed domains, whereas physiological signals, cognitive performance, and communication-based features were less frequently examined, highlighting several underdeveloped but clinically relevant areas. Common measurement approaches included self-report questionnaires and ecological momentary assessment, whilst passive sensing approaches, such as accelerometry, geolocation, device-use metrics, speech features, and actigraphy, captured continuous indicators of sleep–wake cycles, mobility, and activity rhythms. The present synthesis reveals a clear temporal shift: since 2015, the field has transitioned from primarily active, self-report study designs toward multimodal, sensor-based methodologies, driven by advances in smartphones, wearable technologies, and sophisticated data-processing pipelines. Overall, this review highlights substantial progress in digital monitoring for bipolar disorder, with emerging tools offering ecologically valid, continuous assessment of core symptom dimensions. These developments lay essential groundwork for multimodal digital biomarkers with potential applications in early detection, tailored intervention, improved patient outcomes, and longitudinal care.

## Highlights

• Smartphone apps, wearable sensors, and passive monitoring systems are used.
• Across 214 studies, mood, sleep, and activity were measured most frequently.
• Cognition and psychosocial functioning should be more frequently assessed in BD.
• Integrated multimodal datasets broaden the digital markers available for BD.

## Graphical abstract

**Figure:**
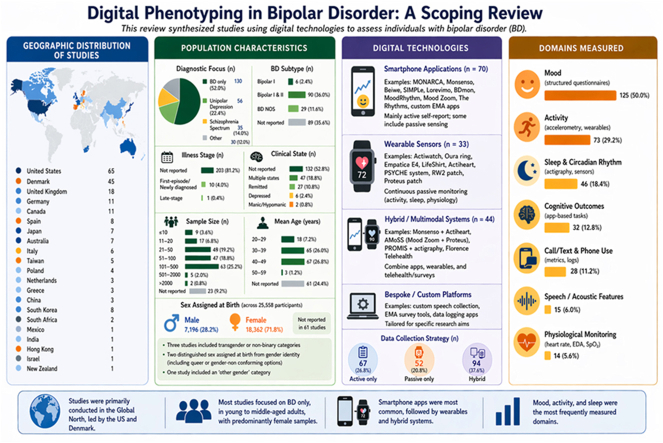

## 1 Introduction

Bipolar disorder (BD) is a complex psychiatric condition characterized by recurrent mood episodes that may involve depressive, manic, or hypomanic states depending on the subtype (American Psychiatric Association, 2013; Singh et al., 2025). This chronic condition is associated with reduced psychosocial functioning and markedly reduced life expectancy, driven by high rates of comorbidities and suicide risk (Chan et al., 2023). Despite advancements in earlier detection and treatment, BD remains challenging to manage due to its heterogeneity, fluctuating symptom course, and high relapse rates that exceed 70% over five years (Marzani et al., 2021). Beyond acute episodes, many individuals experience persistent or residual symptoms that contribute substantially to the burden of illness (Grover et al., 2021), including cognitive deficits, sleep disturbances, and psychosocial dysfunction (Nicoloro-SantaBarbara et al., 2023). As these multidimensional features extend beyond mood polarity and fluctuate over time, they have become key targets for longitudinal assessment. Together, these enduring and dynamic impairments underscore the need for comprehensive follow-up strategies that extend beyond stabilizing acute mood episodes.

Among these features, cognitive impairments and sleep disturbances are particularly prevalent and clinically consequential. Cognitive impairments, including deficits in executive function, memory, and attention, affect 50-70% of individuals with BD. These difficulties often persist during euthymic phases and are strongly associated with poorer occupational and psychosocial outcomes (Nicoloro-SantaBarbara et al., 2023; Boland et al., 2013). Similarly, sleep disturbances, characterized by reduced sleep efficiency and circadian instability, represent a core feature of BD and robustly predict mood episode recurrence (Boland et al., 2013). Together, disruptions of cognition and sleep contribute substantially to functional burden across illness phases and may fluctuate independently of acute mood symptoms. However, brief clinic-based evaluations do not consistently capture these domains or meaningfully distinguish them from mood-related features. This underscores the need for systematic, longitudinal assessment to understand their course and clinical relevance over time (Grover et al., 2021; Boland et al., 2013).

Addressing these ongoing difficulties requires critical monitoring, integrating mood, cognitive, sleep, and psychosocial parameters into treatment planning to optimize care. Measurement-based care (MBC), defined as the systematic use of validated clinical tools to guide treatment decisions, provides a structured approach to achieving this goal (Aboraya et al., 2018). By enabling the systematic monitoring of mood symptoms and related functional outcomes, MBC supports more personalized, targeted, and timely interventions (Aboraya et al., 2018). Indeed, the International Society for Bipolar Disorders (ISBD) has recommended incorporating MBC into routine care for BD to enhance clinical decision-making and improve patient outcomes (Tohen et al., 2009). Despite these recommendations, its adoption remains limited, partly due to a lack of standardized tools and low clinician awareness of existing measures (Cerimele et al., 2019).

Digital technologies extend and operationalize the principles of MBC into everyday life, offering new possibilities for the assessment, monitoring, and management of BD (de Azevedo Cardoso et al., 2024). Unlike traditional clinical-based evaluations, which rely on brief and episodic evaluations, digital tools allow continuous monitoring of symptoms in real-world settings. Smartphones, wearable devices, and other digital tools enable real-time monitoring of mood, sleep, and cognitive performance data. Leveraging these continuous data streams can enhance early identification of relapse risk and inform timely treatment modifications. Ecological momentary assessment (EMA) methods involve prompting individuals multiple times per day to report their current experiences and behaviors in real time and in their usual environments, allowing clinicians and researchers to capture short-term changes and the temporal link between symptoms and contextual factors (Schwartz et al., 2016). For instance, EMA can be used to track mood variability and identify early warning signs of mood episodes in natural settings (Armey et al., 2015). Additionally, digital tools can provide multidimensional assessments by integrating passive data collection methods, such as tracking sleep patterns, physical activity, social interactions, geolocation, and speech patterns. Such digital phenotyping approaches has shown promising early results for identifying mood episodes and informing interventions (de Azevedo Cardoso et al., 2024).

A growing body of research highlights the expanding role of digital technologies in monitoring and managing BD. Importantly, a recent systematic review (Saccaro et al., 2021) found that smartphone apps were widely employed for both active self-monitoring (e.g., mood assessments) and passive data collection (e.g., GPS, accelerometer data), and that several machine-learning models trained on these smartphone features achieved fair to excellent accuracy, including reported classification accuracies as high as 89% for distinguishing depressive from euthymic states and up to 96% precision and 94% recall for detecting mood states (Saccaro et al., 2021). Additionally, wearable sensors and audiovisual tools have emerged as promising technologies, although limitations such as small sample sizes and methodological variability underscore the need for greater standardization and further validation (Saccaro et al., 2021). The findings further indicate that patient compliance with digital monitoring tools is high, with systems such as Monsenso, a smartphone-based self-monitoring system, achieving adherence rates of up to 91% for active and passive monitoring, while mobile sensing data reach compliance levels as high as 99% (Saccaro et al., 2021). These high adherence rates demonstrate the feasibility and acceptability of incorporating digital tools into long-term BD management. Nevertheless, BD remains one of the least explored severe mental illnesses in the context of digital health and MBC (Goldberg et al., 2018). BD is particularly well-suited to benefit from these approaches, given its chronic course, high relapse rates, and the need for ongoing, individualized symptom monitoring across mood, cognitive, sleep, and psychosocial domains. Integrating digital tools into clinical care for BD has the potential to improve clinical and treatment outcomes, while expanding access to care to individuals in underserved contexts.

Despite growing interest in the use of digital tools to support the monitoring and management of BD, the field still lacks a comprehensive overview of the diverse tools and methodologies currently in use. A synthesis of this work is thus necessary to provide clarity on the current digital toolkit available for BD, illuminate critical methodological and conceptual gaps, and guide the development of standardized, evidence-based clinical approaches to support precise monitoring and intervention in this population. The objective of this scoping review was to map the current landscape of digital technologies used to assess and remotely monitor mood, cognitive, sleep, and psychosocial functioning in individuals with BD, and to characterize how these tools have been applied across different study designs and populations.

## 2 Material and methods

### 2.1 Study design

This scoping review was informed by the methodological framework outlined by the Joanna Briggs Institute guidelines for scoping reviews (Peters et al., 2021) and adhered to the reporting guidelines of the Preferred Reporting Items for Systematic Reviews and Meta-Analyses – Extension for Scoping Reviews (PRISMA-ScR) (Tricco et al., 2018). The review was conducted based on a pre-registered protocol, which outlined the objectives, eligibility criteria, search strategy, and data extraction methods: https://osf.io/t4cz3/.

### 2.2 Search strategy and information sources

The search strategy was designed to identify both published and unpublished studies. A systematic, computerized search was conducted across MEDLINE, EMBASE, PsycINFO, and CINAHL, supplemented by gray literature sources such as OpenGrey, as well as clinical trial registries, including ClinicalTrials.gov. The search was last updated on 2026-03-05, without restrictions on language or year of publication. The search terms strategy was structured to capture four primary domain themes: bipolar disorder, assessment tools, digital technology, and dimensions or symptoms assessed (see Supplementary Table 1 for the list of keywords).

### 2.3 Inclusion and exclusion criteria

Selected studies included individuals diagnosed with BD according to the Diagnostic and Statistical Manual of Mental Disorders (DSM; versions III, IV, or 5) or the International Classification of Diseases (ICD; versions 9 or 10) criteria, as well as those identified as at risk (i.e., subsyndromal symptoms and/or first-degree relative with BD). Studies were excluded if they focused on individuals with secondary mania (i.e., induced by external factors, such as medication or thyroid dysregulation). There were no exclusions based on comorbidities, age, gender, or ethnicity, ensuring broad contextual applicability. Studies employing digital tools only for intervention, and not for assessment, were excluded. Eligible sources included peer-reviewed articles and unpublished literature that utilized digital measures for the specified purposes and provided enough details, while conference abstracts and book chapters were excluded.

### 2.4 Study/source of evidence selection

**Fig. 1:**
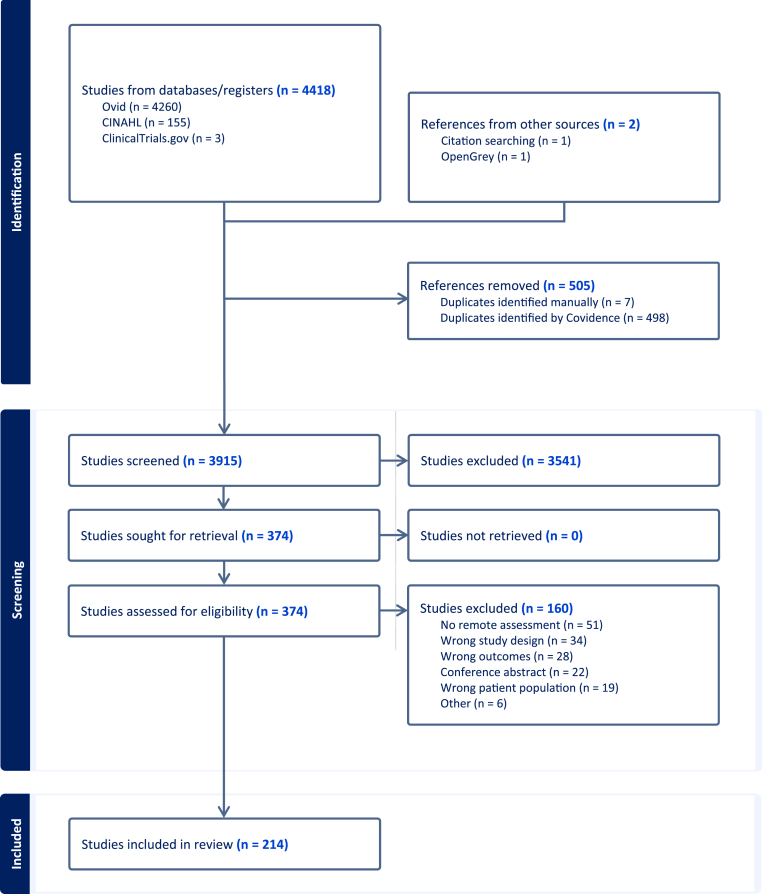
PRISMA flowchart of study selection.

Search results were imported into Covidence systematic review software (Veritas Health Innovation, Melbourne, Australia, available at www.covidence.org), where duplicates were automatically removed. After a pilot test, titles and abstracts were screened independently by two reviewers (HC, ED, SJ, DRC, LS) using predefined inclusion criteria. Full texts of potentially relevant sources were retrieved, and citation details were imported into Covidence. Full-text screening was conducted independently by two reviewers (SJ, CM, DRC, SW), with reasons for exclusion recorded. The search process and study inclusion are detailed in the PRISMA-ScR flow diagram (Fig. 1).

### 2.5 Data extraction

Data extraction was performed using Covidence. A standardized spreadsheet was piloted and refined before completing the data extraction. Quality control was conducted by an independent reviewer (DRC) to ensure data completeness and accuracy. Extracted information included study authors, year of publication, study design, sample characteristics (e.g., age, sex, gender, diagnosis, stage of disorder), and assessment details (e.g., constructs measured, dimensions assessed, psychometric properties, clinical utility).

### 2.6 Analysis

A narrative synthesis was conducted to summarize key findings, identify common themes, and integrate descriptive statistics to illustrate trends in the use of digital tools for BD assessment. Table 1 presents the main data extracted from the included studies, and additional extracted information is provided in Supplementary Table 2.

**Table 1:** Study characteristics.

First author	Year	Country of the study	Platform and/or *sensor* used	Active vs Passive Data	Total N	Diagnoses	BD				
							N	BD Type	Males	Age	
										Mean	SD
										[median]	
Chinman (Chinman et al., 2004)	2004	US	Patient Assessment System (PAS)	Active	91	BD; SSD	45	NR	NR	NR	NR
Bauer (Bauer et al., 2005)	2005	US	ChronoRecord	Active	80	BD	80	BD I; BD II	35	38.67	10.86
Reilly-Harrington (Reilly-Harrington et al., 2010)	2010	US	Interactive Computer Interview for Mania (ICI-M)	Active	100	BD	100	BD I; BD II	40	37.6	NR
Minassian (Minassian et al., 2010)	2010	US	LifeShirt System (VivoMetrics)	Passive	32	BD; SSD	28	NR	15	34.1	13.3
Lieberman (Lieberman et al., 2010)	2010	US	NR	Active	48	BD	48	BD I; BD II; BD NOS	12	39.5 (paper); 35.8 (online)	12.9 (paper); 12.0 (online)
Bauer (Bauer et al., 2011)	2011	US	ChronoRecord	Active	284	BD	284	BD I; BD II; BD NOS	64	37.1	NR
Depp (Depp et al., 2012)	2012	US	NR	Active	40	BD	40	BD I; BD II	16	44.0 (phone); 46.1 (pen & paper)	14.0 (phone); 13.5 (pen & paper)
Miklowitz (Miklowitz et al., 2012)	2012	UK	True Colours system	Active	19	BD	19	BD I; BD II	6	37.2	11.7
Proudfoot (Proudfoot et al., 2012)	2012	Australia	NR	Active	407	BD	407	NR	123	117 under 30 yo	NR
Faurholt-Jepsen (Faurholt-Jepsen et al., 2013)	2013	Denmark	MONARCA	Both	NA	BD	NA	NR	NA	NA	NA
Valenza (Valenza et al., 2013)	2013	Denmark	PSYCHE	Passive	8	BD	8	NR	NR	NR	NR
Faurholt-Jepsen (Faurholt-Jepsen et al., 2014)	2014	Denmark	MONARCA	Both	17	BD	17	BD I; BD II	5	33.4	9.5
Grunerbl (Grunerbl et al., 2015)	2015	Germany	openSMILE	Passive	10	BD	10	NR	NR	NR	NR
Faurholt-Jepsen (Faurholt-Jepsen et al., 2015a)	2015	Denmark	*Actigraph*	Passive	36	BD; DEP	18	NR	7	34.9	12
Faurholt-Jepsen (Faurholt-Jepsen et al., 2015b)	2015	Denmark	MONARCA	Both	61	BD	61	NR	20	29.3	8.4
Faurholt-Jepsen (Faurholt-Jepsen et al., 2015c)	2015	Denmark	MONARCA	Both	67	BD	67	BD I; BD II; BD NOS	22	29.3	8.43
Faurholt-Jepsen (Faurholt-Jepsen et al., 2015d)	2015	Denmark	MONARCA	Both	33	BD	33	BD I; BD II	10	29.1	7.4
Hidalgo-Mazzei (Hidalgo-Mazzei et al., 2015)	2015	Spain	SIMPLe	Both	NA	BD	NA	BD I; BD II	NA	NA	NA
Lanata (Lanata et al., 2015)	2015	Italy	PSYCHE	Passive	10	BD	10	NR	NR	NR	NR
Valenza (Valenza et al., 2015)	2015	Italy	PSYCHE	Passive	8	BD	8	BD I; BD II	NR	NR	NR
Naslund (Naslund et al., 2016)	2016	US	*Fitbit Zip*	Passive	11	BD; DEP; SSD	3	NR	2^ω^	48.2^ω^	11.2^ω^
Abdullah (Abdullah et al., 2016)	2016	US	MoodRhythm	Both	NR	BD	9	BD I; BD II; BD NOS	4	NR	NR
Depp (Depp et al., 2016)	2016	US	NR	Active	41	BD	41	BD I; BD II	19	46.9	11.8
Faedda. (Faedda et al., 2016)	2016	US	NR	Passive	155	BD; Other	48	BD NOS	NR	NR	NR
Kaufmann (Kaufmann et al., 2016)	2016	US	NR	Active	41	BD	41	BD I; BD II	19	46.9	11.8
Schwartz (Schwartz et al., 2016)	2016	US	NR	Active	10	BD	10	BD I; BD II	3	48.94	16.8
Holmes (Holmes et al., 2016)	2016	UK	Mood Action Psychology Programme	Active	14	BD	14	BD I; BD II	2	37	11.82
Tsanas (Tsanas et al., 2016)	2016	UK	Mood Zoom	Active	79	BD; PD	48	NR	16	38	21
Faurholt-Jepsen (Faurholt-Jepsen et al., 2016a)	2016	Denmark	*Actiheart*	Passive	19	BD	19	BD I; BD II	8	[28.1]	IQR: [20.8, 31.3]
Faurholt-Jepsen (Faurholt-Jepsen et al., 2016b)	2016	Denmark	MONARCA	Both	29	BD	29	BD I; BD II	11	30.2	8.8
Faurholt-Jepsen (Faurholt-Jepsen et al., 2016c)	2016	Denmark	MONARCA & openSMILE	Both	28	BD	28	BD I; BD II	10	30.3	9.3
O'Rourke (O'Rourke et al., 2016)	2016	Canada	BD Sx	Active	1010	BD	1010	BD NOS	266	45.28	13.78
Hidalgo-Mazzei (Hidalgo-Mazzei et al., 2016)	2016	Spain	SIMPLe	Active	49	BD	49	BD I; BD II; BD NOS	28	43.92	11.36
Valenza (Valenza et al., 2016)	2016	Italy	PSYCHE	Passive	NR	BD	NR	NR	8	33.43	9.76
Ben-Zeev (Ben-Zeev et al., 2017)	2017	US	NR	Both	27	BD; SSD; Other	9	NR	15^ω^	33^ω^	11.13^ω^
Lobban (Lobban et al., 2017)	2017	UK	NR	Active	96	BD	96	BD I; BD II	37	43.8 (controls) 42 (ERP online)	11.45 (controls); 12.23 (ERP online)
McKnight (McKnight et al., 2017)	2017	UK	True Colours	Active	297	BD	297	BD I; BD II; BD NOS	99	41	13.7
Saunders (Saunders et al., 2017)	2017	UK	True Colours	Active	21	BD	21	BD I; BD II	7	44.38	2.49
Faurholt-Jepsen (Faurholt-Jepsen et al., 2017a)	2017	Denmark	*Actiheart*	Passive	16	BD	16	NR	8	31.3	10.1
Faurholt-Jepsen (Faurholt-Jepsen et al., 2017b)	2017	Denmark	Monsenso	Both	NA	BD; DEP	NA	NR	NA	NA	NA
Kessing (Kessing et al., 2017)	2017	Denmark	Monsenso & *Actiheart*	Both	NA	BD	NA	BD I; BD II	NA	NA	NA
Hidalgo-Mazzei (Hidalgo-Mazzei et al., 2017)	2017	Spain	SIMPLe	Active	51	BD	51	BD I; BD II; BD NOS	28	41(non-completers); 45 (completers)	9 (non-completers); 12 (completers)
Gentili (Gentili et al., 2017)	2017	Italy	PSYCHE	Active	8	BD	8	BD I; BD II	5	40	10
Zulueta (Zulueta et al., 2018)	2018	US	BiAffect	Passive	9	BD	9	BD I; BD II	1	48.67	9.63
Cochran. (Cochran et al., 2018)	2018	US	Lorevimo & *Fitbit Alta HR*	Both	NA	BD	NA	BD I; BD II	NA	NA	NA
Nicholson (Nicholson et al., 2018)	2018	US	WorkingWell mobile support tool	Both	NA	BD; DEP; SSD	NR	NR	NR	NR	NR
Carr (Carr et al., 2018a)	2018	UK	Automated Monitoring of Symptom Severity (AMoSS), Proteus patch & Mood Zoom	Active	129	BD; Other	54	NR	17	39.7	12.6
Carr (Carr et al., 2018b)	2018	UK	Mood Zoom & Proteus Patch	Both	34	BD; PD	20	NR	6	41	11.4
Palmius (Palmius et al., 2018)	2018	UK	NR	Both	37	BD; PD	20	NR	7	Faurholt-Jepsen et al. (2015d)	IQR: 20
Perez Arribas (Perez Arribas et al., 2018)	2018	UK	NR	Passive	130	BD; Other	48	NR	16	38	21
Van den Heuvel (van den Heuvel et al., 2018)	2018	Netherlands	Personal Health Record for Bipolar Disorder (PHR-BD)	Active	66 (T0); 39 (T1)	BD	66 (T0); 39 (T1)	BD I; BD II	44 (T0); 23 (T1)	45.2 (T0); 47.2 (T1)	10.7 (T0); 9.6 (T1)
Muhlbauer (Muhlbauer et al., 2018)	2018	Germany	MovisensXS	Passive	NR	BD	NR	NR	NR	NR	NR
Hidalgo-Mazzei (Hidalgo-Mazzei et al., 2018)	2018	Denmark	SIMPLe	Active	201	BD	201	NR	74	36.59	11
Fletcher (Fletcher et al., 2018)	2018	Australia	NR	Active	300	BD	300	BD NOS	NR	NR	NR
Tanaka (Tanaka et al., 2018)	2018	Japan	*Actiwatch*	Passive	92	BD; DEP	35	BD I; BD II; BD NOS	23	46.9	20.5
Pan (Pan et al., 2018)	2018	China	NR	Passive	21	BD	21	NR	7	34.52	15.32
Anand (Anand et al., 2019)	2019	US	Ginger.io Behavior Platform	Active	35	BD; DEP	6	NR	2	24.3	4.1
Li (Li et al., 2019)	2019	US	NR	Active	10	BD	10	BD I	NR	NR	NR
Merikangas (Merikangas et al., 2019)	2019	US	NR	Both	242	BD; DEP	54	BD I; BD II	NR	48^ω^	16.9
Gordon-Smith (Gordon-Smith et al., 2019)	2019	UK	True Colours	Active	975	BD; SSD	975	BD I; BD II; BD NOS	283	49.7	11.8
Cho (Cho et al., 2019)	2019	South Korea	NR	Passive	55	BD; DEP	37	BD I; BD II	NR	25.92	4.78
Esaki (Esaki et al., 2019)	2019	Japan	*Actiwatch Spectrum Plus*	Passive	175	BD	175	BD I; BD II		45.5	13.1
Faurholt-Jepsen (Faurholt-Jepsen et al., 2019a, 2019b, 2019c, 2019d)	2019	Denmark	MONARCA II	Both	84	BD	84	BD I; BD II	33	43	12.3
Faurholt-Jepsen (Faurholt-Jepsen et al., 2019e)	2019	Denmark	Pulso & Trilogis-Monsenso	Both	59	BD	59	BD I; BD II	19	NR	NR
Zanella-Calzada (Zanella-Calzada et al., 2019)	2019	Mexico	*Actiwatch*	Passive	2112	BD; DEP	NR	NR	NR	NR	NR
Van Til (Van Til et al., 2020)	2020	US	*Fitbit Alta HR wearable*	Both	47	BD	47	BD I; BD II; BD NOS	22	41.9	10.8
Raugh (Raugh et al., 2020)	2020	US	Ilumivu	Both	105	BD; SSD	19	NR	3	37.32	12.82
Choksi (Choksi et al., 2020)	2020	US	mDB	Passive	3	BD	3	NR	NR	NR	NR
Ryan (Ryan et al., 2020)	2020	US	NR	Both	38	BD	26	BD I; BD II; BD NOS	7	46.46	10.55
McGowan (McGowan et al., 2020)	2020	UK	AMoSS &GENEActiv	Both	87	BD; PD	31	BD NOS	10	39.2	12.2
Richter (Richter et al., 2020)	2020	Germany	NR	Active	242	BD; DEP; PD; Other	27	NR	NR	NR	NR
Ebner-Priemer (Ebner-Priemer et al., 2020)	2020	Denmark	BipoSense & ChronoRecord	Both	29	BD	29	BD I; BD II	13	44	11.9
Busk (Busk et al., 2020a, 2020b)	2020	Denmark	Monsenso	Both	84	BD	84	NR	32	43.1	12.4
Faurholt-Jepsen (Faurholt-Jepsen et al., 2020a, 2020b)	2020	Denmark	Monsenso	Both	150	BD	117	BD I; BD II	73	30.9	9.9
Faurholt-Jepsen (Faurholt-Jepsen et al., 2020c)	2020	Denmark	Monsenso	Both	129	BD	129	BD I; BD II	53	43.0 (intervention); 43.2 (control)	12.4 (intervention); 12.4 (control)
Stanislaus (Stanislaus et al., 2020a, 2020b, 2020c)	2020	Denmark	Monsenso	Both	203	BD	203	BD I; BD II	63	28	NR
Cho (Cho et al., 2020)	2020	South Korea	Circadian rhythm of mood (CRM)	Both	73	BD; DEP	53	BD I; BD II	NR	35.30 (test); 22.97 (control)	5.33 (test); 2.86 (control)
Esaki (Esaki et al., 2020)	2020	Japan	*Actiwatch Spectrum Plus & portable photometer (LX-28SD)*	Passive	184	BD	184	BD I; BD II	81	45.1	13.1
Ben-Zeev (Ben-Zeev et al., 2021)	2021	US	CORE	Active	315	BD; DEP; SSD	111	NR	NR	NR	NR
Bomyea (Bomyea et al., 2021)	2021	US	Ecological momentary cognitive testing (EMCT) platform	Active	46	BD	46	NR	16	42.7	11.4
Bowden (Bowden et al., 2021)	2021	US	KIOS	Active	20	BD	20	NR	NR	NR	NR
Jonathan (Jonathan et al., 2021)	2021	US	LiveWell	Active	12	BD	12	BD NOS	4	38	14
Sagorac Gruichich (Sagorac Gruichich et al., 2021)	2021	US	Lorevimo	Active	43	BD	43	BD I; BD II; BD NOS	21	41.58	10.47
Anderson (Anderson et al., 2021)	2021	US	NR	Active	44	BD	44	BD I	16	36.18	12.45
Durand (Durand et al., 2021)	2021	US	NR	Active	173	BD; SSD	71	BD I; BD II	2	39.2	11.75
Harvey (Harvey et al., 2021)	2021	US	NR	Active	173	BD; SSD	71	BD I; BD II	22	39.22	11.75
Jones (Jones et al., 2021)	2021	US	NR	Active	102	BD; SSD	71	BD I; BD II	2	39.22	11.75
Parrish (Parrish et al., 2021a)	2021	US	NR	Active	96	BD; DEP; SSD	16	NR	43^ω^	43.9^ω^	11.2^ω^
Parrish (Parrish et al., 2021b)	2021	US	NR	Active	168	BD; SSD	70	NR	21	39.1	11.9
Ross (Ross et al., 2021)	2021	US	NR	Both	19	BD	11	NR	3	47.09	10.57
Savage (Savage et al., 2021)	2021	US	PROMIS & *Actigraphy*	Passive	98	BD; DEP; SSD; PD	13	BD I	57^ω^	43.67^ω^	12.23^ω^
Gillett (Gillett et al., 2021)	2021	UK	AMoSS	Both	55	BD; PD	17	BD NOS	7	42.24	14.24
Rohricht (Rohricht et al., 2021)	2021	UK	Florence Telehealth System	Active	65	BD; SSD; Other	14	NR	NR	35.2^ω^	NR
So (So et al., 2021)	2021	Hong Kong	NR	Active	64	BD	64	BD I; BD II	15	39.8	12.79
Emden (Emden et al., 2021)	2021	Germany	ReMAP	Both	994	BD; DEP; Other	48	NR	NR	35.99^ω^	13.57^ω^
Faurholt-Jepsen (Faurholt-Jepsen et al., 2021a)	2021	Denmark	Monsenso	Both	98	BD	98	BD I; BD II	47	41.6 (intervention); 43.7 (control)	13.3 (intervention); 13.7 (control)
Faurholt-Jepsen (Faurholt-Jepsen et al., 2021b)	2021	Denmark	Monsenso	Both	129	BD	129	BD NOS	97	46	13
Melbye (Melbye et al., 2021a)	2021	Denmark	Monsenso	Both	105	BD	105	BD I; BD II	26	[21.74]	IQR: 21.28; 22.2
Melbye (Melbye et al., 2021b)	2021	Denmark	Monsenso	Both	40	BD	40		11	[21.6]	IQR: (20.9, 22.3)
Stanislaus (Stanislaus et al., 2021)	2021	Denmark	Monsenso	Both	372	BD; Other	372	BD I; BD II	129	Bauer et al. (2005)	IQR: (24; 36)
Faurholt-Jepsen (Faurholt-Jepsen et al., 2021c)	2021	Denmark	Monsenso & openSMILE	Both	121	BD	121	BD I; BD II	48	35.71	12.35
Miskowiak (Miskowiak et al., 2021)	2021	Denmark	NR	Active	70	BD	35	BD I; BD II	9	31.7	9.0
Ortiz (Ortiz et al., 2021)	2021	Canada	BioModule	Passive	53	BD	53	BD I; BD II	18	44.7	13.1
Farrus (Farrus et al., 2021)	2021	Spain	MoodRecord	Passive	13	BD	13	NR	NR	NR	NR
Anyz (Anyz et al., 2021)	2021	Czech Republic	Mindpax	Active	99	BD	99	NR	39	37.7	11
O'Rourke (O'Rourke et al., 2021)	2021	Canada	BADAS	Active	50	BD	50	NR	NR	NR	NR
Fellendorf (Fellendorf et al., 2021)	2021	Austria	UP!	Both	22	BD	22	NR	12	43.36	10.89
Pellegrini (Pellegrini et al., 2022)	2022	US	Beiwe	Both	41	BD; DEP; SSD	10	BD I; BD II	15^ω^	43^ω^	12^ω^
Dalby (Dalby et al., 2022)	2022	US	NR	Both	24632	BD; DEP	9864	BD I; BD II	1864	32.1	7.8
Fortuna (Fortuna et al., 2022)	2022	US	NR	Both	30 total (21 completed)	BD; DEP; SSD; Other	9	NR	NR	NR	NR
Moore (Moore et al., 2022)	2022	US	NR	Active	46	BD	45	NR	15	43	12
Russell (Russell et al., 2022)	2022	US	NR	Active	376	BD; SSD	173	BD I; BD II	57	40.5 (in person); 37.8 (remote)	11.7 (in person); 11 (remote)
Titone (Titone et al., 2022)	2022	US	NR	Active	70	BD	70	BD I; BD II	25	47.4	9.17
Lynham (Lynham et al., 2022)	2022	UK	TestMyBrain	Active	65 (validation); 887 (web-based)	BD; DEP; SSD; Other	16 (validation); 146 (web-based)	BD I; BD II	5	51.1	13.8
Bennett (Bennett et al., 2022)	2022	South Korea	BiAffect	Passive	291	BD	NA	NR	192^ω^	41.3^ω^	NR
Kang (Kang et al., 2022)	2022	South Korea	Search Your Mind	Both	NA	BD; DEP; Other	NA	NR	NA	NA	NA
Bos (Bos et al., 2022)	2022	Netherlands	RoQua	Active	20	BD	20	BD I; BD II	4	NR	NR
Daus (Daus et al., 2022)	2022	Germany	NR	Both	3	BD	3	BD I; BD II	2	37.67	11
Faurholt-Jepsen (Faurholt-Jepsen et al., 2022a)	2022	Denmark	Monsenso	Both	218	BD; DEP	98	BD NOS	59	44.1	13.27
Stanislaus et al.	2022	Denmark	Monsenso	Both	223	BD	223	BD I; BD II	73	Chinman et al. (2004)	IQR: (Land et al., 2019; Massaro et al., 2025; Kushniruk et al., 2024; Chen et al., 2022; Nierenberg et al., 2023; Chinman et al., 2004; Bauer et al., 2005, 2011; Reilly-Harrington et al., 2010; Minassian et al., 2010; Lieberman et al., 2010; Depp et al., 2012; Miklowitz et al., 2012; Proudfoot et al., 2012)
Faurholt-Jepsen (Faurholt-Jepsen et al., 2022b)	2022	Denmark	Monsenso & OpenSmile	Both	169	BD; DEP	121	NR	48	35.7	12.3
Ortiz (Ortiz et al., 2022)	2022	Canada	*Oura Ring & eVAS*	Passive	NA	BD	NA	BD I; BD II	NA	NA	NA
Michalak (Michalak et al., 2022)	2022	Canada	PolarUs	Active	NA	BD	NA	BD I; BD II; BD NOS	NA	NA	NA
Braund (Braund et al., 2022)	2022	Australia	Socialise	Both	121	BD; DEP	42	NR	23	41	13.16
Tseng (Tseng et al., 2022)	2022	Taiwan	NR	Both	159	BD	159	NR	70	34.5	11.34
Lee (Lee et al., 2022)	2022	South Korea	NR	Both	270	BD; DEP	175	BD I; BD II	123^ω^	23.3	3.63
Dominiak (Dominiak et al., 2022)	2022	Poland	BDmon	Both	51	BD	51	BD I; BD II	23	36.2	9.5
Koga (Koga et al., 2022)	2022	Japan	myBeat WHS-1	Passive	19	BD; DEP	6	NR	7^ω^	42.3^ω^	12.5^ω^
Sigurdardottir (Sigurdardottir et al., 2022)	2022	Iceland	DataWell digital health platform	Both	18	BD; SSD	7	NR	NR	NR	NR
Zlatintsi (Zlatintsi et al., 2022)	2022	Greece	*E-Prevention System & S3 Frontier Smartwatch*	Passive	24	BD; SSD; Other	8	BD I	16^ω^	30.8^ω^	6.56^ω^
Goulding (Goulding et al., 2023)	2023	US	LiveWell	Active	205	BD	205	BD I	80	42	12
Cochran (Cochran et al., 2023)	2023	US	Lorevimo	Active	30	BD	30	BD I; BD II	12	42.7	11.11
Weintraub (Weintraub et al., 2023)	2023	US	MyCoachConnect (FFT-MCC) and FFT-Track	Passive	44	BD; DEP	8	BD I; BD II; BD NOS	NR	NR	NR
Dalkner (Dalkner et al., 2023)	2023	US	NR	Active	240	BD; SSD	114	BD I; BD II	36	38.45	11.69
Liu (Liu et al., 2023)	2023	US	NR	Active	36	BD	36	NR	0	40.7 (cycling); 56.0 (menopause)	7.9 (cycling); 4.0 (menopause)
Fisher (Fisher et al., 2023)	2023	US	PRIME	Both	100	BD; DEP; SSD; Other	10	NR	NR	NR	NR
Nduka et al.	2023	UK	OCOsense	Passive	84	BD; DEP	NR	NR	NR	NR	NR
Lewis (Lewis et al., 2023)	2023	UK	True Colours	Active	649	BD	649	BD I; BD II	187	52	NR
Reininghaus (Reininghaus et al., 2023)	2023	Germany	NR	Active	92	BD; DEP; SSD; Other	NR	NR	NR	NR	NR
Faurholt-Jepsen (Faurholt-Jepsen et al., 2023a)	2023	Denmark	Monsenso	Both	NA	BD	NA	NR	NA	NA	NA
Faurholt-Jepsen (Faurholt-Jepsen et al., 2023b, 2023c)	2023	Denmark	Monsenso	Both	374	BD; DEP	316	NR	102	38.5	10.8
Ortiz (Ortiz et al., 2023)	2023	Canada	*Oura Health Oy*	Passive	87	BD	87	BD I; BD II	28	38.9	12.4
Esaki (Esaki et al., 2023)	2023	Japan	*Actiwatch Spectrum Plus (Respironics) & LX-28SD (Sato Shoji)*	Passive	194	BD	194	BD I; BD II	90	[44.0]	IQR: (35.7-53.0)
Nakagome (Nakagome et al., 2023)	2023	Japan	*Fitbit Sense*	Passive	110	BD; DEP; SSD; Other	15	NR	7	43.5	10.7
Kalisperakis (Kalisperakis et al., 2023)	2023	Greece	NR	Passive	35	BD; SSD	15	BD I; BD II	9	33.4	6.75
Liu (Liu et al., 2024)	2024	US	BiAffect	Passive	101	BD; DEP	14	BD I; BD II	NR	33.1^ω^	6.58^ω^
Lipschitz (Lipschitz et al., 2025)	2024	US	*Fitbit Inspire*	Passive	54	BD	54	BD I; BD II	14	40.3	13.9
Moran (Moran et al., 2024)	2024	US	*Garmin Vivosmart 4 fit watch and Crosscheck app*	Both	313	BD; DEP; SSD	61	NR	21	37.23	8.61
Larsen (Larsen et al., 2024)	2024	US	Mental Fitness	Active	104	BD; DEP; PD; Other	2	NR	NR	NR	NR
Langholm (Langholm et al., 2024)	2024	US	mindLAMP	Both	84	BD; DEP	NR	BD I; BD II	NR	NR	NR
Paquin (Paquin et al., 2024)	2024	US	NR	Active	409	BD; SSD	220	BD I; BD II	69	NR	NR
Cochran (Cochran, 2024)	2024	US	*Reusable wearable sensor version 2 (RW2) patc*		60	BD; DEP; SSD	12	BD I	NR	NR	NR
Urosevic et al.	2024	US	*VA mPRO, FollowMee & Recorder Plus or ASR Voice Recorder*	Both	NA	BD	NA	BD I; BD II; BD NOS	NA	NA	NA
Corponi (Corponi et al., 2024)	2024	Spain	*Empatica E4 wristband*	Both	75	BD; DEP	63	NR	NR	NR	NR
Bayas (Bayas et al., 2024)	2024	Germany	NR	Both	NA	BD; PD; Other	NA	NR	NA	NA	NA
Faurholt-Jepsen (Faurholt-Jepsen et al., 2025)	2024	Denmark	Monsenso	Passive	106	BD; DEP	47	NR	23	41.6	13.3
Faurholt-Jepsen (Faurholt-Jepsen et al., 2024)	2024	Denmark	Monsenso	Both	138	BD; DEP	64	BD I; NR	25	44.1	13.27
Stokholm (Stokholm et al., 2024)	2024	Denmark	Monsenso	Both	NA	BD	NA	NR	NA	NA	NA
Von Hofacker (von Hofacker et al., 2024)	2024	Denmark	Monsenso	Both	258	BD	258	BD I; BD II	82	30	9.15
Li (Li et al., 2024)	2024	Canada	MoodFX	Active	49	BD; DEP	11	NR	NR	NR	NR
Mao (Mao et al., 2024)	2024	Canada	NR	Active	1004	BD; DEP; SSD; PD; Other	206	NR	79	NR	NR
Halabi (Halabi et al., 2024)	2024	Canada	*Oura smart sensor*	Both	145	BD	145	BD I; BD II	53	38.1	11.9
Davis (Davis et al., 2024)	2024	Australia	NR	Active	NA	BD; DEP	NA	NR	NA	NA	NA
Moraga (Moraga et al., 2024)	2024	The Netherlands	RoQua	Active	20	BD	20	BD I; BD II	4	NR	NR
Lee (Lee et al., 2024)	2024	Taiwan	Beiwe	Both	28	BD; DEP	11	NR	4	44.09	9.97
Hsu (Hsu et al., 2024)	2024	Taiwan	MoodSensing	Both	181	BD	181	NR	78	36.6	NR
Anmella (Anmella et al., 2024a)	2024	Spain	*E4 Empatica wristband*	Passive	NA	BD; DEP	NA	NR	NA	NA	NA
Anmella (Anmella et al., 2024b)	2024	Spain	*Empatica E4 wearable device*	Passive	49	BD	49	BD I; BD II; BD NOS	22	47	14
Yeom (Yeom et al., 2024)	2024	South Korea	Circadian Rhythm for Mood	Both	NA	BD; DEP	NA	BD I; BD II	NA	NA	NA
Lim (Lim et al., 2024)	2024	South Korea	*Fitbit Charge HR*	Passive	168	BD; DEP	101	BD I; BD II	93^ω^	23.2 (recurrences); 24.3 (no recurrences)	3.38 (recurrences); 4.24 (no recurrences)
Song (Song et al., 2024)	2024	South Korea	NR	Both	139	BD; DEP	94	BD I; BD II	36	24.7 (I); 23.0 (II)	4.37 (I); 2.74 (II)
Kaczmarek-Majer (Kaczmarek-Majer et al., 2025)	2024	Poland	BDmon	Passive	51	BD	51	BD I; BD II	23	35	NR
Ksiazek (Ksiazek et al., 2024)	2024	Poland	*Polar H10 chest strap*	Passive	30	BD; SSD	7	NR	14^ω^	41.43^ω^	13.06^ω^
Mordechai et al.	2024	Israel	Datos Health	Both	NA	BD; DEP; SSD; PD	NA	NR	NA	NA	NA
Rajitha (Rajitha et al., 2024)	2024	India	NR	Passive	100	BD	100	BD I; BD II	NR	34.9	12
Ikaheimonen (Ikaheimonen et al., 2024)	2024	Finland	MoMo-Mood	Both	164	BD; DEP; PD	21	NR	3	37.1	10.3
Luo (Luo et al., 2024)	2024	China	NR	Active	198	BD; DEP; SSD; Other	61	NR	23	27.1	9.4
Zhu (Zhu et al., 2024)	2024	China	NR	Both	75	BD; DEP	26	NR	NR	NR	NR
Lynch (Lynch et al., 2025)	2025	US	Aktibipo Self-Rating Questionnaire (ASERT)	Active	61	BD	61	BD I; BD II	17	39.3	14
Culbreth (Culbreth et al., 2025)	2025	US	Beiwe	Both	136	BD; DEP; SSD	47	NR	17	36.57	8.04
Ajilore (Ajilore et al., 2025)	2025	US	BiAffect	Both	18	BD	18	BD I; BD II	6	47.4	10.6
Ning (Ning et al., 2025)	2025	US	BiAffect	Passive	95	BD; DEP; Other	14	BD I; BD II; BD NOS	47	32.4	−4.82
Lipschitz (Lipschitz et al., 2025)	2025	US	*Fitbit Inspire*	Both	54	BD	54	BD I; BD II	14	40.3	13.9
Epperson (Epperson et al., 2025)	2025	US	Rhythms	Passive	104	BD; DEP; Other	23	BD I; BD II	32^ω^	42.1^ω^	15.9^ω^
Lee (Lee et al., 2025)	2025	Taiwan	Beiwe	Both	47	BD; DEP	20	NR	8	40.35	11.15
Wu (Wu et al., 2025)	2025	Taiwan	*Garmin Vivosmart* 4 & MEDGOD	Both	24	BD	24	NR	9	38	9
Valenzuela-Pascual (Valenzuela-Pascual et al., 2025)	2025	Spain	*E4 wrist-bands from Empatica*	Passive	102	BD	102	NR	41	47.33	NR
Kaczmarek-Majer (Kaczmarek-Majer et al., 2025)	2025	Poland	BDmon	Both	51	BD	51	BD I; BD II	23	36	NR
Glastad (Glastad et al., 2025)	2025	Norway	MinDag	Active	32	BD	32	BD I; BD II; BD NOS	13	33.28	10.1
Min (Min et al., 2025)	2025	Korea	NR	Passive	92	BD	92	BD II; BD NOS	22	29.28; 31.97	9.53; 12.45
Kurihara (Kurihara et al., 2025)	2025	Japan	*Actiwatch Spectrum*	Passive	47	BD	47	NR	21	45.7	15.1
Crocamo (Crocamo et al., 2025)	2025	Italy	SPEAKapp	Passive	32	BD	32	NR	16	49.6	14.3
Garyfalli et al. (Garyfalli et al., 2025)	2025	Greece	NR	Passive	38	BD; SSD	15	NR	26	30.5	7.3
Clemens (Clemens et al., 2025)	2025	Germany	BipoSense	Both	27	BD	27	NR	7	46	12.3
Ludwig (Ludwig et al., 2025a)	2025	Germany	movisensXS	Both	28	BD	28	BD I; BD II	12	43.82	12.09
Ludwig (Ludwig et al., 2025b)	2025	Germany	movisensXS	Both	29	BD	29	BD I; BD II	13	43.97	11.9
Aledavood (Aledavood et al., 2025)	2025	Finland	MoMo-Mood: AWARE app & *Actigraphy*	Both	121	BD; DEP; Other	21	NR	NR	NR	NR
Zarp (Zarp et al., 2025)	2025	Denmark	Internet-based Cognitive Assessment Tool (ICAT)	Both	31	BD	31	BD I; BD II	18	28	NR
Faurholt-Jepsen (Faurholt-Jepsen et al., 2025)	2025	Denmark	Monsenso	Both	106	BD; DEP	47	NR	23	41.6	13.3
Pfaffenseller (Pfaffenseller et al., 2025)	2025	Canada	Mindpax	Both	20	BD	20	BD I; BD II; BD NOS	NR	NR	NR
Ortiz (Ortiz et al., 2025)	2025	Canada	*Oura Ring (Gen 2)*	Passive	50	BD	50	BD I; BD II	19	33.9	9.9
Ratheesh (Ratheesh et al., 2025)	2025	Australia	Bipolar Early Intervention Using New Digital Technologies (BLEND)	Active	21	BD	21	BD I; BD II	3	2.03	2.31
Morton (Morton et al., 2025)	2025	Australia	PolarUs app	Active	25	BD	25	BD I; BD II; BD NOS	10	38.3	11.5
Halabi (Halabi et al., 2026)	2026	Canada	*Oura Ring*	Both	133	BD	133	BD I; BD II	50	38.2	11.9

*Notes.* BD: bipolar disorders; DEP: major depressive disorders; SSD: schizophrenia-spectrum disorders; NR: not reported; PD: personality disorders; UK: United Kingdom; US: United States of America; ^ω:^ whole group.

## 3 Results

**Fig. 2:**
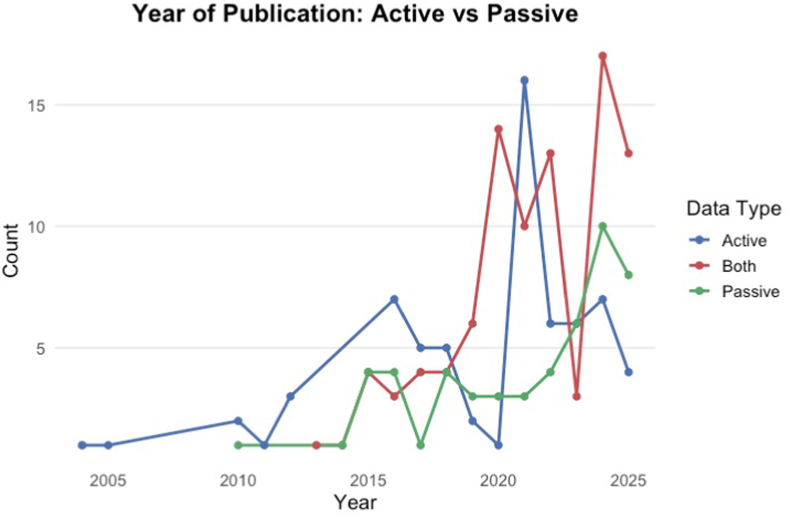
Temporal Trends in Publications by Data Collection Type (Active, Passive, and Hybrid) An overview of publication trends over time. The figure displays the annual number of studies published between 2003 and 2025 employing active (self-report), passive (sensor-based), or hybrid (combined) data collection approaches.

In total, 4418 references were identified across databases and registers, including Ovid (n = 4260), CINAHL (n = 155), ClinicalTrials.gov (n = 3), citation searching (n = 1), and OpenGrey (n = 1). Following the removal of 505 duplicates (498 by Covidence and 7 manually) and conference abstracts (n = 21), 3915 titles and abstracts were screened. Of these, 374 articles were sought for full-text retrieval. After a detailed assessment, 160 articles were excluded (see Fig. 1 for the PRISMA flow diagram). In total, 214 studies met the inclusion criteria and were included in this review. These studies were conducted during the period January 2004–March 2026 (Fig. 2 reports the number of studies per year until 2025). Between 2004 and 2012, publication activity remained low, with only 1–3 studies assessing active modalities per year and virtually no passive or hybrid (i.e., integrating active and passive) approaches. The first study to include a hybrid approach appeared in 2013. From 2015 onward, the number of publications increased to 8 studies in 2015 (4 passive, 4 hybrid) and 14 studies in 2016 (7 active, 4 passive, 3 hybrid). Publication counts continued to rise after 2018, with 29 studies in 2021 (15 active, 3 passive, 10 hybrid), a peak of 34 in 2024 (7 active, 10 passive, 17 hybrid), 25 in 2025 (4 active, 8 passive, 13 hybrid), and one hybrid paper so far in 2026. Across the full period, active approaches accounted for 67 studies, hybrid approaches for 94, and passive-only approaches for 52.

### 3.1 Geographic distribution of studies

**Fig. 3:**
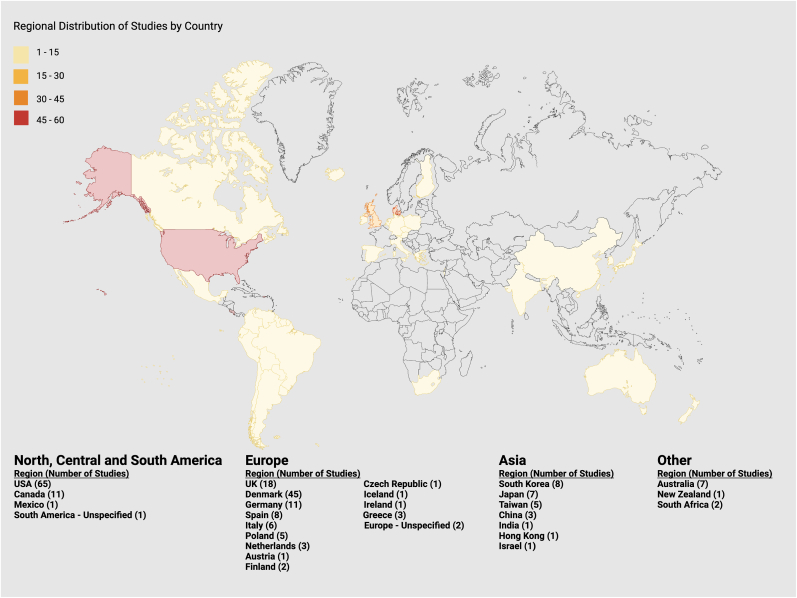
Regional distribution of studies by country Heat map illustrating the geographic origin of all studies included in the review (n = 214). Each country is shaded according to the number of studies conducted, with darker colours representing higher counts.

The included studies were conducted across a wide range of countries, most of which were conducted in the Global North regions. As shown in Fig. 3, the largest number of studies were conducted in the United States (n = 65) and Denmark (n = 45), followed by the United Kingdom (n = 18) and Germany (n = 11). Other European countries contributed smaller but notable proportions, including Spain (n = 8), Italy (n = 6), the Netherlands (n = 3), Poland (n = 4), and Greece (n = 3). In North America, besides the US, the included studies were conducted in Canada (n = 11) and Mexico (n = 1). A small number of studies were conducted in Asia, most commonly in Japan (n = 7), South Korea (n = 8), China (n = 3), and Taiwan (n = 5), with additional contributions from India (n = 1), and Hong Kong (n = 1). Studies were also identified in Australia (n = 7), New Zealand (n = 1), Israel (n = 1), and South Africa (n = 2).

### 3.2 Population characteristics

Most studies focused exclusively on individuals with BD (n = 130). Smaller groups examined transdiagnostic populations, including unipolar depression (n = 56), schizophrenia spectrum disorders (n = 35), and other psychiatric conditions (n = 30).

Where BD subtypes were specified, 6 studies examined bipolar I, 90 studies included bipolar I and II, and 29 studies included bipolar disorder not otherwise specified (NOS). BD subtype was not reported in 89 studies. Most studies (n = 203) did not report the illness stage; 10 studies included participants with first-episode psychosis or newly diagnosed bipolar disorder, and only 1 study focused on late-stage illness. Moreover, some studies included participants during specific mood states; 6 studies included participants in a depressive state, 2 studies included manic or hypomanic participants, 27 studies included remitted samples, and 47 studies described multiple clinical states. However, most studies (n = 132) did not provide information on the clinical state.

Sample sizes varied considerably across studies. Nine studies included ten or fewer participants, 17 studies recruited between 11 and 20 participants, and 48 studies reported sample sizes of 21–50. Moderate-sized cohorts were most common, with 47 studies enrolling 51–100 participants and 63 enrolling 101–500 participants. Larger samples were less frequent: 5 studies recruited 501–2000 participants, and only 2 studies exceeded 2000 participants. Twenty-three studies didn't report sample size (protocols were included in the review).

Age distributions of individuals with BD generally reflected young to middle adulthood (mean: 38.4 ± 7.20 years). 18 studies reported mean ages in the 20–29 range, 65 studies reported mean ages in the 30–39 range, and 67 studies reported mean ages in the 40–49 range. Only 3 studies reported mean ages in the 50–59 range, while 61 studies did not report participant age.

Across all studies, data on sex assigned at birth were collected for 25558 participants with BD, of whom 7196 were male (28.2%), and 18362 were female (71.8%). Across the included studies, three reported transgender or non-binary categories, two distinguished sex assigned at birth from gender identity (including queer or gender-non-conforming options and ‘prefer not to disclose’), and one study included an ‘other gender’ category. Sex distribution was not reported in 61 studies.

### 3.3 Digital technologies

The most frequently used technologies were smartphone applications (n = 70). Examples included the MONARCA system (and derivatives such as MONARCA I, II, and app variations), the Monsenso app/system, the Beiwe app, and the SIMPLe 1.0/2.0 applications. Other apps reported were Lorevimo, BDmon, MoodRhythm, Mood Zoom (MZ), The Rhythms app, and custom-built ecological momentary assessment (EMA) systems designed for data logging or survey delivery. A small number of studies used established web-based platforms such as True Colours or third-party systems like Ginger.io. These applications primarily supported active self-report of mood, symptoms, or functioning, though several incorporated passive sensing (e.g., phone usage, speech features).

Wearable sensors such as actigraphy watches, smart rings, and physiological patches (n = 33) represented the second major category. Frequently reported devices included Actiwatch and related actigraphy watches (e.g., Spectrum Plus models), the Oura smart ring, and the Empatica E4 wristband. Physiological monitoring platforms such as the LifeShirt system (VivoMetrics), the Actiheart device, and the PSYCHE system (t-shirt with ECG, respiration, and posture sensors) were also employed. In addition, patch-based technologies such as the RW2 wearable sensor and Proteus digital patch were used in selected studies, often paired with mobile apps for integrated monitoring.

Hybrid and multimodal systems were also represented (n = 44). Some studies combined app-based platforms with wearables, such as Monsenso with Actiheart, or the AMoSS system (Mood Zoom app plus Proteus patch). Others reported mixed systems that paired telehealth or survey tools with actigraphy (e.g., PROMIS with actigraphy, Florence Telehealth).

Finally, a small proportion of studies employed bespoke platforms, including custom smartphone speech collection systems, tailored EMA survey tools, and research-specific data logging apps. These systems typically served specialized research aims such as acoustic analysis, mobility tracking, or feasibility testing.

Smartphones were generally deployed for both active self-report and passive sensing, whereas wearables were primarily used for continuous passive monitoring of activity, sleep, and circadian rhythm. In terms of overall data collection strategy, 67 studies relied exclusively on active methods, 52 on passive monitoring, and 94 on hybrid.

### 3.4 Domains measured

Mood was by far the most common target, often captured through structured questionnaires (n = 125). Sleep and circadian rhythm measures were also commonly evaluated (n = 46), typically through passive monitoring such as actigraphy-based monitoring of circadian rhythm. Activity levels were similarly well represented (n = 73), typically derived from accelerometry or wearable sensors. Physiological monitoring was less widespread but still present in a notable subset (n = 14), capturing signals such as heart rate, electrodermal activity, or blood oxygen saturation. Cognitive outcomes (e.g., attention, memory, executive functioning) were also studied, with only 32 studies including app-based cognitive tasks. Similarly, psychosocial and communication-related domains were underrepresented: speech or acoustic features (n = 15), call logs and text messaging or phone use metrics (n = 28) appeared in only a minority of studies. Table 2 shows all the platforms/systems that have been presented in more than two studies.

**Table 2:** Description of the main platforms presented in more than two publications.

Platform/system name		Monsenso	MONARCA	BiAffect	MovisensXS	PSYCHE	True Colours	Beiwe app	SIMPLe	AMoSS	ChronoRecord	Lorevimo app	BDmon app
Number of studies		32	8	5	5	5	5	4	4	3	3	3	3
[References]		See Table 1	(Faurholt-Jepsen et al., 2013, 2014, 2015b, 2015c, 2015d, 2016b, 2016c, 2019a, 2019b, 2019c, 2019d)	(Zulueta et al., 2018; Bennett et al., 2022; Liu et al., 2024; Ajilore et al., 2025; Ning et al., 2025)	(Muhlbauer et al., 2018; Ludwig et al., 2025a, 2025b)	(Valenza et al., 2013, 2015, 2016; Lanata et al., 2015; Gentili et al., 2017)	(Miklowitz et al., 2012; McKnight et al., 2017; Saunders et al., 2017; Gordon-Smith et al., 2019; Lewis et al., 2023)	(Pellegrini et al., 2022; Lee et al., 2024, 2025; Culbreth et al., 2025)	(Hidalgo-Mazzei et al., 2015, 2016, 2017, 2018)	(Carr et al., 2018a; McGowan et al., 2020; Gillett et al., 2021)	(Bauer et al., 2005, 2011; Ebner-Priemer et al., 2020)	(Cochran et al., 2018, 2023; Sagorac Gruichich et al., 2021)	(Dominiak et al., 2022; Kaczmarek-Majer et al., 2025)
**PASSIVE**													

*Wearable*				*X*	*X*	*X*	*X*	*X*		*X*^*a*^			
*Smartphone*		*X*	*X*	*X*	*X*	*X*			*X*	*X*			*X*

Domains (number)		8	4	2	7	4	1	2	3	5	0	3	5

Physiological Sensors	HR					X				X		X^b^	
Sleep and Circadian Sensors	Light exposure	X				X				X		X^b^	
Activity	Movement		X	X	X	X	X	X	X	X^a^		X^b^	
	(3-D accelerometer or gyroscope)												
	Step count	X			X								
	GPS	X			X			X					X
Phone use	Screen on/off timings	X			X								X
	Keyboard use			X									
	App use				X				X				
Number of messages sent/received		X	X		X					X			X
Call logs		X	X		X^c^				X	X			X
Acoustic features		X	X			X							X
Other		X											

**ACTIVE**													

*App*		*X*	*X*		*X*^*c*^			*X*	*X*			*X*	*X*
*Website*		*X*				*X*	*X*			*X*	*X*		
*Link*							*X*						

Domains (number)		6	5	0	3	2	2	1	5	2	1	3	1

Questionnaire	Mood assessment	X	X		X^c^	X	X	X	X	X	X	X	X
	Other symptoms	X	X						X	X^a^		X^b^	
	Functioning	X							X				
	Cognition	X	X										
	Sleep	X	X		X^c^	X	X		X			X^b^	
	Other	X	X		X^c^				X				

*Notes.* Superscripts letters refer to the combination of the presented platform/system and another named here: ^a^GENEActiv; ^b^Fitbit Alta HR; ^c^m-Path Sense & Sensor;^d^Mood Zoom app;^e^AMoSS and Mood Zoom app.

## 4 Discussion

The objective of this scoping review was to identify and describe the range of digital tools currently available to monitor the various dimensions affecting individuals living with bipolar disorder (BD). This comprehensive review demonstrates that, particularly over the past decade, there has been substantial progress in the development of digital monitoring methods, evolving from initially active methods to increasingly more passive and hybrid ones, designed to assess specific features of BD, primarily mood and sleep. Most of these developments have originated from studies conducted in high-income countries, relying predominantly on smartphone applications and, increasingly, wearable sensors. Participant recruitment across studies covered a broad spectrum of individuals with BD, although many samples were overwhelmingly skewed toward female participants, with women representing over than two-thirds of the BD sample. In addition, the included studies tended to enroll relatively young individuals.

### 4.1 Temporal trends in data collection methods

Studies employed a broad range of digital measures spanning active and passive modalities. Active approaches most often involved self-report questionnaires, ecological momentary assessment, or app-based cognitive tasks, typically targeting mood, sleep quality, energy, and daily functioning. Passive methods relied on background sensing, including accelerometry, geolocation, phone usage patterns, speech and voice features, and actigraphy, providing continuous information on sleep, activity, and mobility in real-world settings. While some studies relied exclusively on active or passive data, many integrated both, generating multimodal datasets that combined subjective experience with objective behavioural markers. Since 2015, digital data collection in BD has accelerated, driven by these hybrid designs that integrate active and passive monitoring, while purely active methods have plateaued, while purely passive approaches have shown a steady and meaningful rise, contributing substantially to the overall growth in publications. This trend reflects a shift in the field toward the more sensor-based, objective methodologies, as digital technologies now enable the continuous and objective tracking of behavioural and physiological information through smartphones and wearable devices (de Azevedo Cardoso et al., 2023). Such systems allow near real-time quantification of mood, activity, and circadian patterns, supporting ongoing monitoring and providing greater insight into the course of the illness (Alam et al., 2025). Many mobile platforms incorporate self-reported mood assessments, which remain central to the diagnosis and clinical management of BD. In parallel, recent studies have increasingly integrated passive sensor data to complement self-report measures by capturing behavioral and physiological correlates of mood in real-world settings. Self-report and passive sensing approaches serve distinct but complementary purposes. Self-reported measures provide direct access to subjective mood experience, which remains fundamental to psychiatric diagnosis and treatment planning. In contrast, passive sensor data may offer continuous, context-rich behavioral information that can help identify patterns, early warning signs, or state transitions that may not be immediately apparent to patients or clinicians. Rather than replacing self-report, passive monitoring may enhance longitudinal understanding when integrated within a multimodal assessment framework. This distinction is particularly relevant in BD, where diagnostic classification is fundamentally based on reported subjective experience rather than solely on observable behavioral markers.

Despite advances, several major challenges remain. Variability in sensor hardware, sampling frequency, and data-processing pipelines still hinders reproducibility and comparability across studies (Alam et al., 2025). Data integration is also impeded by the lack of common metadata standards (i.e., information describing the characteristics of data), inconsistent annotation methods, and fragmented regulatory frameworks for health data sharing (Alam et al., 2025). Moreover, ethical and privacy concerns persist, particularly around the passive collection of geolocation and physiological signals, which may inadvertently disclose sensitive personal information (Eysenbach et al., 2021). Alam et al. (2025) further emphasize that the field still lacks validated pipelines for feature extraction and harmonization across devices, underscoring the need for standardized application programming interfaces and open-source reference datasets to ensure scientific transparency. Thus, while the transition to sensor-based digital phenotyping has expanded the scope and granularity of BD assessment, its long-term clinical utility will depend on addressing issues of standardization, interpretability, and data governance before such technologies can be seamlessly integrated into routine MBC. Nevertheless, the decreasing reliance on purely active, self-reported data may reflect a broader shift toward integrating multimodal and objective measurement strategies in contemporary research and clinical settings.

### 4.2 Geographic distributions of studies

While digital monitoring methods in BD have increasingly shifted from active to passive or hybrid methods, these trends have been documented inconsistently across the globe. The geographic distribution of included studies demonstrates a concentration in the Global North (including here North America, Europe, Japan, South Korea, Israel, Australia, and New Zealand). Specifically, most studies were conducted in North America and Western Europe. In contrast, relatively few studies were conducted in the Global South. This concentration raises important questions regarding accessibility and cultural adaptability, both in terms of the technological infrastructure required for digital data collection and the linguistic and socioeconomic barriers that may limit participation in such research.

However, beyond access and infrastructure, cultural safety and representativeness must be prioritized in the development and implementation of digital monitoring tools. Individuals diagnosed with BD experience symptoms within cultural frameworks shaped by beliefs, values, and norms (Fullerton et al., 2025). For instance, emotional expression patterns of BD vary significantly between East Asian, African, and Western cultures, while spiritual and societal beliefs in regions of West Africa and the Middle East affect help-seeking behaviours and treatment adherence (Dei-Asamoa et al., 2025). Furthermore, cultural biases embedded within symptom rating scales and diagnostic criteria contribute to misdiagnosis and under-recognition of diverse symptom manifestations, underpinning the importance of culturally adapted diagnostic instruments and interventions. Without careful cultural calibration, digital monitoring tools developed in alignment with historic Eurocentric diagnostic criteria and symptom expression risk systematically under-recognize manifestations that are more common in other cultural settings or misclassifying them as atypical or subthreshold. Such misalignments fundamentally contribute to the misdiagnosis or under-recognition of symptom presentations that are only legible within Eurocentric diagnostic frameworks. Consequently, there is an urgent need for future work to develop culturally adapted measurement frameworks using culturally and ancestrally diverse participatory design approaches to move beyond mere technical synchronization of these tools. Without such representation, digital monitoring tools will lack the predictive value and clinical relevance required to serve the global populations they hope to extend to.

In regions with limited access to specialized healthcare or digital infrastructure, future research should focus on creating wearable and smartphone-based systems that are both technically adaptable and economically sustainable, and follow the REASSURED framework (Real-time connectivity; Ease of data collection; Affordable; Sensitive, Specific; User-friendly; Rapid and robust; simple; Environmentally friendly; Deliverable to end-users) (Land et al., 2019). Low-cost wearable sensors capable of capturing basic physiological parameters (e.g., actigraphy, heart rate, sleep metrics) can be integrated with existing smartphone platforms to reduce reliance on proprietary devices (Massaro et al., 2025). Similarly, applications that enable offline data capture with delayed synchronization would allow continuous monitoring in settings with unstable internet connectivity. Moreover, AI-based translation and natural language processing tools could facilitate multilingual interfaces, allowing mobile applications and digital assessments to be accessible across languages and literacy levels (Kushniruk et al., 2024). Adoption of open source software and other open science practices (e.g., co-design with lived experience experts, open hardware) would promote inclusivity and collaboration in digital mental health. In addition, embedding speech and text recognition systems that support regional dialects or non-Western languages would broaden engagement in populations historically underrepresented (Kushniruk et al., 2024).

### 4.3 Population recruited and future directions

Across studies, reporting of participant characteristics was limited, with illness stage and clinical state often incompletely described. More than half of the studies did not specify whether the participants were euthymic, depressed, or manic at the time of assessment. This is a potential confounding factor that can markedly influence engagement with the digital tools. However, open inclusion criteria in terms of mood state may also be perceived as more inclusive and representative of the BD population. Similarly, the stage of illness was rarely detailed, preventing comparison of digital markers across early, stable, and chronic periods of the illness. Consequently, it remains unclear whether identified digital features represent enduring traits or state-dependent fluctuations.

Additionally, sample sizes were highly variable, ranging from small pilot cohorts to a limited number of large-scale studies, which reduces the robustness and generalizability of findings. Smaller samples also increase the likelihood of selection bias and hinder the development of reliable, externally validated algorithms for MBC (Chen et al., 2022). Demographic representation was similarly uneven: most cohorts consisted of young to middle-aged adults, with minimal inclusion of older individuals, who may differ in digital literacy, technology adoption, and circadian rhythm profiles. As a result, current evidence may not fully reflect the real-world feasibility of digital monitoring across diverse populations, including those less familiar with smartphone or wearable technologies. Sex imbalance was another consistent limitation. Across the included studies that reported sex proportion, men accounted for only 22.72% of participants, despite BD affecting both men and women relatively equally (Nierenberg et al., 2023). This underrepresentation limits the ability to explore potentially sex-specific behavioural or physiological signatures and the generalizability of digital phenotyping outcomes.

By systematically mapping these demographic and methodological trends, this review helps clarify where evidence is concentrated and where critical gaps remain, particularly when focusing on illness stage, clinical state, and demographic diversity. These challenges are common when conducting research with clinical populations, but digital monitoring is uniquely positioned to improve them due to its flexibility. Future studies should address these gaps and confounders by recruiting larger and more demographically balanced cohorts, explicitly defining and reporting clinical and illness stages, and expanding inclusion to older adults and underrepresented groups. Longitudinal and cross-diagnostic designs will be essential to disentangle trait-versus state-related digital features and to strengthen the translational value of digital tools for BD. In addition, some of the domains were less explored, notably cognition and psychosocial functioning which are central dimensions and should be more frequently assessed in BD.

### 4.4 Limitations

By design, this scoping review aimed to provide a broad overview rather than a quantitative synthesis. The heterogeneity of study designs, outcomes, and populations limits direct comparison, and no formal quality appraisal was performed, consistent with scoping review methodology, which prioritizes mapping the breadth of evidence rather than critically evaluating study quality. However, the absence of quality assessment should be interpreted cautiously, particularly given that reporting standards varied substantially across studies. Notably, more than half of the included studies did not clearly specify participants’ clinical mood state at the time of assessment, which represents a significant methodological limitation. Given the central importance of mood state in BD, the lack of this information may reflect weaknesses in study design or reporting and limits the interpretability and comparability of digital markers across investigations.

The processes of screening, data extraction, and domain classification were conducted by multiple reviewers, which may have introduced biases, despite training and oversight by a coordinating reviewer. Only peer-reviewed articles and accessible protocols were included, which may have introduced publication bias and excluded relevant but unpublished or ongoing digital monitoring projects. Our review might minimize the reality of ongoing developments in the Global South, where scientific outputs are more frequently disseminated in local or regional settings. Finally, the heterogeneity of reporting, especially regarding illness stage, clinical state, and demographic information, further limited comparability across studies. Despite these constraints, this review offers a comprehensive synthesis of the current state of digital monitoring research in BD and highlights critical conceptual, methodological, demographic, and technological gaps to inform future research.

## 5 Conclusions

In conclusion, this scoping review highlights the rapid expansion of digital technologies applied to BD. Advances in wearable and smartphone-based technologies have enabled real-time, continuous assessment and tracking of mood, activity, sleep, and functioning with increasing precision. With the advances of artificial intelligence and machine learning models, these developments represent an important step toward precise, data-driven methods in research and clinical practice. Hence, digital tools can provide a foundation for earlier detection of mood episodes, individualized intervention, and improved longitudinal care for people with BD. Future research should prioritize validation across diverse populations and illness stages to ensure their reliability, generalizability, and equitable implementation. In addition, establishing standardized frameworks for data collection, feature extraction, and validation will be essential to ensure reproducibility and applicability.

## Funding

This research did not receive any specific grant from funding agencies in the public, commercial, or not-for-profit sectors. KML, GS, and DRC are supported by salary awards through the Fonds de Recherche du Québec Santé (#367856, #330282, and #367157, respectively).

## Declaration of competing interest

The authors declare the following financial interests/personal relationships which may be considered as potential competing interests: Delphine Raucher-Chene reports a relationship with Otsuka Canada that includes: speaking and lecture fees. Katie M. Lavigne reports a relationship with Otsuka Canada, Lundbeck Canada, and Boehringer Ingelheim that includes: speaking and lecture fees. The other authors declare that they have no known competing financial interests or personal relationships that could have appeared to influence the work reported in this paper.
