# Supplementary material for: Digital tools for assessing bipolar disorder: A scoping review of the current landscape
Source: Neurosci Appl. 2026 Apr 21;5:107004. doi: 10.1016/j.nsa.2026.107004 (PMC13140024; doi:10.1016/j.nsa.2026.107004)
Supplement: Multimedia component 1 [file mmc1.docx]

**Digital Tools for Assessing Bipolar Disorder: A Scoping Review of the Current Landscape**

**Supplementary Table 1.** Search strategy used for the scoping review at the initial search and follow-up search

| Domains | Search terms |
| --- | --- |
| Psychiatric condition | (bipolar disorder* OR manic-depressive psychos* OR manic depressive psychos* OR mania OR manic OR bipolar depression OR mixed features) AND |
| Assessment | (scale* OR measure* OR questionnaire* OR survey* OR assessment* OR evaluat* OR test* OR self-report OR self report OR self-rated OR self rated OR self rating OR self-rating OR clinician-rated OR clinician rated OR passive data OR health data OR psychometr*) AND |
| Digital | (remote* OR online* OR mobile* OR digital* OR computer* OR electronic health record OR wearable OR sensor) AND |
| Dimensions assessed | ((severity ADJ3 illness) OR symptom* OR insight OR clinical OR functioning OR functional OR recovery OR daily living OR social OR psychosocial OR psychological OR occupation* OR job OR employ* OR activit* OR wellbeing OR well-being OR (quality ADJ3 life) OR QoL OR (life ADJ3 satisfaction) OR impairment OR disability OR cogniti* OR neuropsych* OR neurocogniti* OR percepti* OR executive function* OR learning OR memory OR mental process* OR mental status OR (processing ADJ3 speed) OR reasoning OR verbal fluency OR attention OR (theory ADJ3 mind) OR emotion*) |

*Notes.* ADJ3: words are adjacent within 3 words

**Supplementary Table 2.** Additional information extracted from included studies.

| **Study ID** | **Platform and/or sensor used** | **Language** | **Digital technology** | **Active vs Passive** |
| --- | --- | --- | --- | --- |
| Chinman 2004 | Patient Assessment System (PAS) | English | website | Active |
| Bauer 2005 | ChronoRecord | English | other | Active |
| Reilly-Harrington 2010 | Interactive Computer Interview for Mania (ICI-M) | English | app | Active |
| Lieberman 2010 | NR | English | online survey | Active |
| Minassian 2010 | LifeShirt System (VivoMetrics) | English | sensor | Passive |
| Bauer 2011 | ChronoRecord | English | other | Active |
| Proudfoot 2012 | NR | English | website; online survey | Active |
| Miklowitz 2012 | True Colours system | English | online survey | Active |
| Depp 2012 | NR | NR | app | Active |
| Faurholt-Jepsen 2013 | MONARCA | Danish | app | Both |
| Faurholt-Jepsen 2014 | MONARCA | Danish | app | Both |
| Valenza 2014 | PSYCHE | Italian | sensor | Passive |
| Faurholt-Jepsen 2015 | MONARCA | Danish | app; sensor | Both |
| Faurholt-Jepsen 2015 | Actigraph | Danish | sensor | Passive |
| Faurholt-Jepsen 2015 | MONARCA | Danish | app | Both |
| Faurholt-Jepsen 2015 | MONARCA | Danish | app | Both |
| Grunerbl 2015 | MONARCA | German | app | Passive |
| Lanata 2015 | PSYCHE | Italian | app; sensor | Passive |
| Valenza 2015 | PSYCHE | Italian | sensor | Passive |
| Hidalgo-Mazzei 2015 | SIMPLe | Spanish | app | Both |
| Faurholt-Jepsen 2016 | Actiheart | None | sensor | Passive |
| Faurholt-Jepsen 2016 | MONARCA | Danish | app | Both |
| Faurholt-Jepsen 2016 | MONARCA and openSMILE | Danish | app; sensor | Both |
| Valenza 2016 | PSYCHE | Italian | sensor | Passive |
| Hidalgo-Mazzei 2016 | SIMPLe | Spanish | app | Active |
| Holmes 2016 | Mood Action Psychology Programme | English | website | Active |
| Tsanas 2016 | Mood Zoom | English | app | Active |
| Abdullah 2016 | MoodRhythm | English | app; sensor | Both |
| Schwartz 2016 | NR | English | app | Active |
| Naslund 2016 | Fitbit Zip | English | sensor | Passive |
| Kaufmann 2016 | NR | English | online survey | Active |
| Depp 2016 | NR | English | online survey | Active |
| Faedda 2016 | NR | English | sensor | Passive |
| O'Rourke 2016 | BD Sx | English | app | Active |
| Kessing 2017 | Monsenso and Actiheart | Danish | app | Both |
| Faurholt-Jepsen 2014 | Monsenso | Danish | app | Both |
| Faurholt-Jepsen 2017 | Actiheart | NA | sensor | Passive |
| Faurholt-Jepsen 2017 | Monsenso | Danish | app; sensor | Both |
| Gentili 2017 | PSYCHE | Italian | app; sensor | Active |
| Hidalgo-Mazzei 2017 | SIMPLe | Spanish | app | Active |
| Saunders 2017 | True Colours | English | app | Active |
| Lobban 2017 | NR | English | website | Active |
| McKnight 2017 | True Colours | English | online survey | Active |
| Ben-Zeev 2017 | NR | English | sensor; other | Both |
| Fletcher 2018 | NR | English | online survey | Active |
| Muhlbauer 2018 | MovisensXS | German | app; sensor | Passive |
| vandenHeuvel 2018 | Personal Health Record for Bipolar Disorder (PHR-BD) | Dutch | website; online survey | Active |
| Hidalgo-Mazzei 2018 | SIMPLe | Spanish | app | Active |
| Pan 2018 | NR | Cantonese | other | Passive |
| Tanaka 2018 | Actiwatch | NA | sensor | Passive |
| Palmius 2018 | NR | English | app; sensor | Passive |
| Carr 2018 | Automated Monitoring of Symptom Severity (AMoSS), Proteus patch and Mood Zoom | English | app; sensor | Both |
| PerezArribas 2018 | NR | English | app | Active |
| Carr 2018 | Mood Zoom and Proteus Patch | English | app; sensor | Both |
| Gordon-Smith 2019 | True Colours | English | website; online survey; other | Active |
| Nicholson 2018 | WorkingWell mobile support tool | English | app | Both |
| Cochran 2018 | Lorevimo and Fitbit Alta HR | English | app | Both |
| Faurholt-Jepsen 2019 | MONARCA II | Danish | app | Both |
| Faurholt-Jepsen 2019 | Monsenso | Danish | app | Both |
| Faurholt-Jepsen 2019 | Monsenso | Danish | app | Both |
| Faurholt-Jepsen 2019 | Monsenso | Danish | app | Both |
| Faurholt-Jepsen 2019 | Pulso & Trilogis-Monsenso | Danish, Italian, Spanish | app; sensor | Both |
| Esaki 2019 | Actiwatch Spectrum Plus | Japanese | sensor | Passive |
| Cho 2019 | NR | NA | sensor | Passive |
| Zanella-Calzada 2019 | Actiwatch | NA | sensor | Passive |
| Anand 2019 | Ginger.io Behavior Platform | English | app | Active |
| Li 2019 | NR | English | app | Active |
| Merikangas 2019 | NR | English | sensor | Both |
| Faurholt-Jepsen 2020 | Monsenso | Danish | app | Both |
| Stanislaus 2020 | Monsenso | Danish | app | Both |
| Faurholt-Jepsen 2020 | Monsenso | Danish | app | Both |
| Busk 2020 | Monsenso | Danish | app | Both |
| Stanislaus 2020 | Monsenso | English | app | Both |
| Busk 2020 | Monsenso | Danish | website; app | Both |
| Faurholt-Jepsen 2020 | Monsenso | Danish | app | Both |
| Stanislaus 2020 | Monsenso | Danish | app | Both |
| Ebner-Priemer 2020 | BipoSense | German |  | Both |
| Richter 2020 | NR | German | online survey | Active |
| Esaki 2020 | Actiwatch Spectrum Plus & portable photometer (LX-28SD) | NA | sensor | Passive |
| Cho 2020 | Circadian rhythm of mood (CRM) | Korean | app | Both |
| McGowan 2020 | NR | English | sensor | Both |
| Zulueta 2018 | BiAffect | English | app; sensor | Passive |
| Choksi 2020 | mDB | English | app; sensor | Passive |
| Raugh 2020 | Ilumivu | English | app; sensor | Both |
| Ryan 2020 | NR | English | app | Both |
| VanTil 2020 | Fitbit Alta HR wearable | English | app; sensor | Both |
| Ortiz 2021 | BioModule | English | sensor | Passive |
| Fellendorf 2021 | UP! | German | app; sensor | Both |
| Anyz 2021 | Mindpax | English; German; Czech | app | Active |
| Stanislaus 2021 | Monsenso | Danish | app | Both |
| Melbye 2021 | Monsenso | Danish | app; sensor | Both |
| Melbye 2021 | Monsenso | Danish | app; sensor | Both |
| Faurholt-Jepsen 2021 | Monsenso & openSMILE | Danish | app | Both |
| Faurholt-Jepsen 2021 | Monsenso | Danish | app | Both |
| Emden 2021 | ReMAP | German | app | Both |
| Farrus 2021 | MoodRecord | Not language-dependent (acoustic features only) | app | Passive |
| Faurholt-Jepsen 2021 | Monsenso | Danish | app; sensor | Both |
| Miskowiak 2021 | NR | Danish | online survey | Active |
| So 2021 | NR | Cantonese | online survey | Active |
| Gillett 2021 | AMoSS | English | app | Both |
| Rohricht 2021 | Florence Telehealth System | English | other | Active |
| Harvey 2021 | NR | English | website; app | Active |
| Ben-Zeev 2021 | CORE | English | online survey | |
| SagoracGruichich 2021 | Lorevimo | English | app | Active |
| Bowden 2021 | KIOS | English | app | Active |
| Anderson 2021 | NR | English | online survey | Active |
| Ross 2021 | NR | English | app | Both |
| Jonathan 2021 | LiveWell | English | app | Active |
| Parrish 2021 | NR | English | app; other | Active |
| Bomyea 2021 | Ecological momentary cognitive testing | English | app | Active |
| Parrish 2021 | (EMCT) platform | English | app | Active |
| Jones 2021 | NR | English | other | Active |
| Durand 2021 | NR | English | online survey; other | Active |
| Savage 2021 | PROMIS & Actigraphy | English | sensor | Passive |
| O'Rourke 2021 | BADAS | English | app | Active |
| Braund 2022 | Socialise | English | app | Both |
| Ortiz 2022 | Oura Ring + eVAS | English | sensor | Passive |
| Michalak 2022 | PolarUs | English | app | Active |
| Stanislaus 2022 | Monsenso | Danish | app | Both |
| Faurholt-Jepsen 2022 | Monsenso & OpenSmile | Danish | app | Both |
| Zlatintsi 2022 | E-Prevention System & S3 Frontier Smartwatch | Greek | app; sensor | Passive |
| Sigurdardottir 2022 | DataWell digital health platform | NR | app; sensor | Both |
| Bos 2022 | RoQua | Dutch | online survey | Active |
| DAUS 2022 | NR | German | app | Both |
| Dominiak 2022 | BDmon | NR | app | Both |
| Faurholt-Jepsen 2022 | Monsenso | Danish | app; sensor | Both |
| Koga 2022 | myBeat WHS-1 | Japanese | sensor | Passive |
| Kang 2022 | Search Your Mind | Korean | app | Both |
| Tseng 2022 | NR | Cantonese | app | Both |
| Lee 2022 | NR | Korean | other | Both |
| Lynham 2022 | TestMyBrain | English | website | Active |
| Dalby 2022 | NR | English | website; online survey | Both |
| Pellegrini 2022 | Beiwe | English | app | Both |
| Titone 2022 | NR | English | online survey | Active |
| Russell 2022 | NR | English | website | Active |
| Fortuna 2022 | NR | English | app | Both |
| Moore 2022 | NR | English | app; online survey | Active |
| Bennett 2022 | BiAffect | English | app; sensor | Passive |
| Ortiz 2023 | Oura Health Oy | NR | app; sensor | Passive |
| Faurholt-Jepsen 2023 | Monsenso | NR | app | Both |
| Faurholt-Jepsen 2023 | Monsenso | Danish | app; sensor | Both |
| Reininghaus 2023 | NR | German | app | Active |
| Kalisperakis 2023 | NR | NR | sensor | Passive |
| Faurholt-Jepsen 2023 | Monsenso | NR | app | Both |
| Nakagome 2023 | Fitbit Sense | Japanese | sensor | Passive |
| Nduka 2023 | OCOsense | English | sensor | Passive |
| Lewis 2023 | True Colours | English | website; other | Active |
| Weintraub 2023 | MyCoachConnect (FFT-MCC) and FFT-Track | English | app | Passive |
| Goulding 2023 | LiveWell | English | app | Active |
| Cochran 2023 | Lorevimo | English | app | Active |
| Liu 2023 | NR | English | online survey; other | Active |
| Dalkner 2023 | NR | English | online survey | Active |
| Esaki 2023 | Actiwatch Spectrum Plus (Respironics) and LX-28SD (Sato Shoji) | Korean | sensor | Passive |
| Davis 2024 | NR | English | online survey | Active |
| Li 2024 | MoodFX | English; French | online survey | Active |
| Mao 2024 | NR | English | online survey | Active |
| Halabi 2024 | Oura smart sensor | English | sensor | Both |
| Faurholt-Jepsen 2024 | Monsenso | Danish | app | Both |
| Faurholt-Jepsen 2024 | Monsenso | Danish | app | Both |
| vonHofacker 2024 | Monsenso | Danish | app | Both |
| Stokholm 2024 | Monsenso | Danish | app | Both |
| Ikaheimonen 2024 | MoMo-Mood | Finish | app | Both |
| Bayas 2024 | NR | German | app; sensor | Both |
| Kaczmarek-Majer 2024 | Bdmon | NR | app | Passive |
| Ksiazek 2024 | Polar H10 chest strap | Polish | sensor | Passive |
| Anmella 2024 | Empatica E4 wearable device | Spanish | sensor | Passive |
| Anmella 2024 | E4 Empatica wristband | NR | sensor | Passive |
| Corponi 2024 | Empatica E4 wristband | NA | sensor | Passive |
| Moraga 2024 | RoQua | Dutch | online survey | Active |
| Zhu 2024 | NR | Cantonese | app | Both |
| Luo 2024 | NR | Cantonese | website; online survey | Active |
| Rajitha 2024 | NR | Indian | sensor | Passive |
| Mordechai 2024 | Datos Health | Hebrew | app | Both |
| Song 2024 | NR | Korean | sensor; online survey | Both |
| Lim 2024 | Fitbit Charge HR | English | sensor | Passive |
| Yeom 2024 | Circadian Rhythm for Mood | | app | Both |
| Lee 2024 | Beiwe | Cantonese | sensor; other | Both |
| Hsu 2024 | MoodSensing | Cantonese; English | app | Both |
| Liu 2024 | BiAffect | NA | sensor | Passive |
| Moran 2024 | Garmin Vivosmart 4 fit watch and Crosscheck app | NR | app; sensor; online survey | Both |
| Urosevic 2024 | VA mPRO, FollowMee, and Recorder Plus or ASR Voice Recorder | English | app | Both |
| Larsen 2024 | Mental Fitness | English | app | Active |
| Langholm 2024 | mindLAMP | English | app | Both |
| Lipschitz 2024 | Fitbit Inspire | | sensor | Passive |
| Paquin 2024 | NR | English | online survey | Active |
| Fisher 2023 | PRIME | English | app | Both |
| Cochran 2024 | Reusable wearable sensor version 2 (RW2) patc | English | sensor | Passive |
| Ortiz 2025 | Oura Ring (Gen 2) | English | sensor | Passive |
| Epperson 2025 | Rhythms | English | app | Passive |
